# Role of barium enema examination for the diagnosis of submucosal invasion depth in T1 colorectal cancers

**DOI:** 10.1186/s40644-021-00437-z

**Published:** 2021-12-07

**Authors:** Keisuke Kawasaki, Takehiro Torisu, Takahisa Nagahata, Motohiro Esaki, Koichi Kurahara, Makoto Eizuka, Yoshihito Tanaka, Minako Fujiwara, Shinichiro Kawatoko, Yumi Oshiro, Shun Yamada, Koji Ikegami, Shin Fujioka, Yuta Fuyuno, Yuichi Matsuno, Junji Umeno, Tomohiko Moriyama, Takanari Kitazono, Tamotsu Sugai, Takayuki Matsumoto

**Affiliations:** 1 Department of Medicine and Clinical Science, Graduate School of Medical Sciences, Kyushu University, 3-1-1, Maidashi, Higashi-ku, Fukuoka, 812-0054 Japan; 2 Division of Gastroenterology, Department of Internal Medicine, Iwate Medical University, Yahaba, 028-3695 Japan; 3 Division of Gastroenterology, Department of Internal Medicine, Faculty of Medicine, Saga University, Saga, 849-8501 Japan; 4 Division of Gastroenterology, Matsuyama Red Cross Hospital, Matsuyama, 790-8524 Japan; 5 Department of Diagnostic Pathology, Iwate Medical University, Yahaba, 790-8524 Japan; 6 Department of Pathology, National Hospital Organization Kyushu Medical Center, Fukuoka, 810-8563 Japan; 7 Department of Anatomic Pathology, Graduate School of Medical Sciences, Kyushu University, Fukuoka, 812-0054 Japan; 8 Department of Pathology, Matsuyama Red Cross Hospital, Matsuyama, 790-8524 Japan

**Keywords:** Barium enema, Colorectal cancer, Invasion depth, Cancer, CT colonography

## Abstract

**Background:**

The indication for endoscopic resection for submucosally invasive colorectal cancer (T1-CRC) depends on the preoperative diagnosis of invasion depth. The aim of this investigation was to evaluate the association between barium enema examination (BE) profile views and depth of submucosal (SM) invasion in CRCs.

**Methods:**

We reviewed the radiographic and endoscopic findings of 145 T1-CRCs diagnosed from 2008 to 2019. We measured the widths of horizontal and vertical rigidity under a BE profile view corresponding to CRC and compared the values with SM invasion depth. Horizontal rigidity was defined as the horizontal length and vertical rigidity as the vertical width of the barium defect corresponding to each target lesion. The most appropriate cut-off values for predicting SM invasion ≥1.8 mm were calculated by receiver operating characteristic curve analysis.

**Results:**

Values of horizontal rigidity (*r* = 0.626, *P* < 0.05) and vertical rigidity (*r* = 0.482, *P* < 0.05) correlated significantly with SM invasion depth. The most appropriate cut-off values for the prediction of SM invasion depth ≥ 1.8 mm were 4.5 mm for horizontal rigidity, with an accuracy of 80.7%; and 0.7 mm for vertical rigidity, with an accuracy of 77.9%. The prevalence of lympho-vascular invasion was significantly different when those cut-off values were applied (43.2% vs. 17.5% for horizontal rigidity, *P* < 0.005).

**Conclusions:**

In T1-CRC, values of horizontal and vertical rigidities under a BE profile view were correlated with SM invasion depth. While the accuracy of the rigidities for the prediction of SM invasion depth ≥ 1.8 mm was not high, horizontal rigidity may be predictive of lympho-vascular invasion, thus aiding in therapeutic decision-making.

## Background

Endoscopic resection (ER), such as endoscopic submucosal dissection (ESD) and endoscopic mucosal resection (EMR), has become widely accepted as a treatment of choice for colorectal epithelial neoplasms [1, 2]. According to recent guidelines, ER is indicated for high-grade dysplasia or early cancer with minimal submucosal invasion [3, 4]. However, lymph node metastasis occurs at a rate of approximately 10% among patients with surgically resected submucosally invasive (T1-) colorectal cancer (CRC). Pathologic risk factors for lymph node metastasis after complete ER are submucosal (SM) invasion depth more than 1 mm, tumor histology (poor differentiation, signet-ring cell or mucinous carcinoma), lympho-vascular invasion, and tumor budding (grade 2/3) [5–9]. According to the 2019 guidelines proposed by the Japanese Society for Cancer of the Colon and Rectum (JSCCR), additional surgery coupled with lymph node dissection has been suggested for T1-CRC with any one or more risk factor(s) after ER [3, 8]. In contrast, Nakadoi et al. [5] showed that T1-CRCs with SM invasion depth < 1.8 mm without any of the above-mentioned risk factors had not metastasized. More recently, Park et al. [10] showed that SM invasion depth > 2.5 mm was associated with a high recurrence rate.

Barium enema examination (BE), CT-colonography and colonoscopy are the main procedures used to diagnose the invasion depth of CRC. While wall rigidity under the BE profile view has been shown to be predictive of invasion depth in CRC, the use of BE has been declining in recent years [11–13]. Alternatively, magnifying narrow-band imaging colonoscopy (M-NBI) and magnifying chromoendoscopy (MCE) have become more widely used for the diagnosis of colorectal epithelial neoplasms, since both procedures can be applied easily to target lesions during conventional colonoscopy, and they have high accuracy for the diagnosis of deep submucosal invasion [14–20]. M-NBI enables clear visualization of the microvascular architecture and surface structure of colorectal tumors [14]. With the use of MCE, crypt openings on the surface of the tumors, referred to as the pit pattern, can be classified into various types [15, 16].

To date, however, few studies have compared the association between SM invasion depth and wall rigidity under BE in T1-CRC. We thus conducted a retrospective analysis to examine the association between tumor invasion depth and wall rigidity under a BE profile view in T1-CRCs. We also investigated the findings of M-NBI and MCE in T1-CRCs.

## Methods

### Study population

The current investigation was based on retrospective data collection. We reviewed the colonoscopy database coupled with histology at our institutions from 2008 to 2019 and identified all patients with a diagnosis of T1-CRC removed endoscopically or surgically. ER was performed for colorectal lesions with a pretreatment diagnosis of up to minimal submucosal invasion, while surgery was the first choice for those with deep submucosal invasion. Among those T1-CRCs, we excluded cancers that were not evaluated by BE, M-NBI and MCE. The protocol of the present study was approved by the Institutional Review Board at each of our institutions.

Consequently, we enrolled 145 lesions in 145 patients (male/female, 77/68; mean age, 69.2 ± 9.5 years) that were treated with ER (EMR; *n* = 7, ESD; *n* = 22) or surgery (colectomy with LN dissection; *n* = 114, transanal minimally invasive surgery without lymph node dissection; *n* = 2). The mean size of the lesions was 20.4 ± 14.8 mm. There were 39 lesions in the right side of the colon (cecum to transverse colon) and 106 lesions in the left side of the colon (descending colon to rectum). Endoscopically, 75 lesions appeared as the protruding type, and 70 lesions as the superficial type based on the Paris classification [21].

### BE and colonoscopic evaluation

**Fig. 1:**
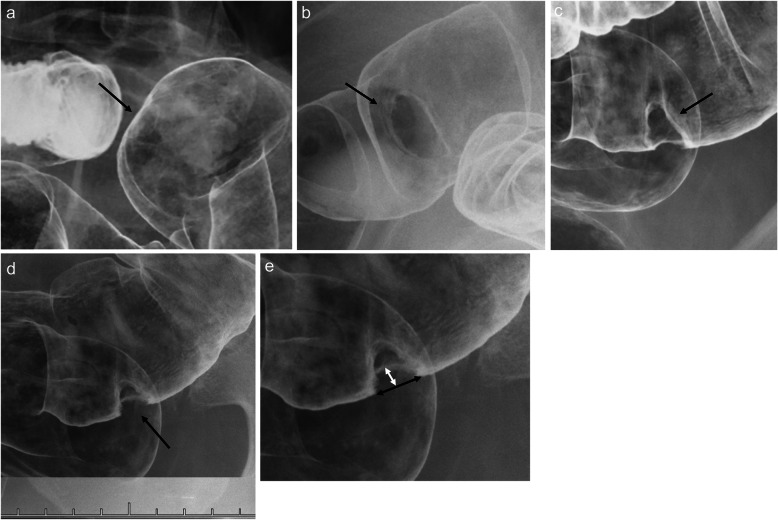
Images of barium enema examination. **a**: Wall rigidity under the profile view at the lesion in the sigmoid colon is absent (arrow). Values of horizontal and vertical rigidities are 0 mm. **b**: The enface view. There is a lesion of the protruding type in the sigmoid colon (arrow). **c**: The oblique view. The lesion in Fig. 1b is gradually moved (arrow). **d**: The profile view. Wall rigidity under the profile view at the lesion in Fig. 1b is present (arrow). **e**: High-power view of Fig. 1d. The horizontal rigidity is 13.3 mm (black arrow), and the vertical rigidity is 4.4 mm (white arrow)

BE was performed with a double-contrast technique using 100% wt./vol. barium sulphate by gastroenterologists who were experts in BE. For each CRC, the enface and profile views and in-between views were taken during BE under substantial luminal extension. In accordance with the procedures published by Watari et al. [13] and Matsumoto et al. [22], the presence of wall rigidity under the profile view at the site of CRC was reviewed by the observers. Also, we measured the horizontal and vertical rigidities under the BE profile view at the site of CRC. We defined horizontal rigidity as the horizontal length and vertical rigidity as the vertical width of the barium defect corresponding to each target lesion (Fig. 1). For lesions without rigidity, the value was measured as 0 mm.

For the evaluation of findings obtained by M-NBI and MCE, the Japan NBI Expert Team (JNET) classification and the pit pattern classification were applied. The JNET classification consists of four types according to superficial vessel and surface patterns (1, 2A, 2B and 3), while the pit pattern under MCE was classified into eight types (I, II, III_L_, IIIs, IV, V_I_ low-irregularity, V_I_ high-irregularity and V_N_) [14–16]. It has been shown that type 3 of the JNET classification and the pit patterns of V_I_ high-irregularity and V_N_ are signs of invasion depth ≥ 1 mm.

Each of the BE, M-NBI and MCE images was evaluated by two observers. Both observers were experts with experience in gastroenterological radiology and endoscopy for 16 years and nine years, respectively. They were blinded to the histopathologic findings, and they separately assessed the radiologic and endoscopic images of each lesion. In cases in which interpretations of BE findings of the presence of wall rigidity under the profile view or endoscopic findings were discordant, the two gastroenterologists discussed the images until a common consensus was obtained. When CRCs had wall rigidity under the BE profile view, the observers immediately measured the horizontal length and the vertical width of the barium defect at the site of CRC. The former was regarded as the horizontal rigidity and the latter as the vertical rigidity.

### Histopathologic evaluation

Three pathologists, who are experts in gastrointestinal histopathology, diagnosed T1-CRCs independently according to the classification reported by the World Health Organization in 2019 [23]. The analyzed pathological items included histologic type, depth of SM invasion, lympho-vascular invasion, tumor budding, and lymph node metastasis. The depth of SM invasion was measured according to the JSCCR guidelines [3]. When it was possible to clearly identify the muscularis mucosa or to outline provisional lines for the muscularis mucosa, the depth of SM invasion was measured from the lower border of the muscularis mucosa. In cases of obscure lines for the muscularis mucosa, the depth of SM invasion was measured from the surface of the lesion. CRCs were further classified as those with invasion depth ≥ 1.8 mm and those with invasion depth < 1.8 mm in accordance with the cut-off value reported by Nakadoi et al. [5]. The cut-off value has been shown to be predictive of lymph node metastasis in T1-CRCs. Tumor budding was defined as a single cancer cell or cancer clusters with < 5 cancer cells observed in the invasive frontal region, and was classified as follows: grade 1, < 5 budding foci; grade 2, 5 to 9 budding foci; or grade 3, ≥10 budding foci [9, 24, 25].

### Statistical analysis

Parametric data are expressed as mean ± standard deviation (SD). Nonparametric data are expressed as numbers and percentages. Comparisons between any two groups were performed with the Mann–Whitney test or chi-square test where appropriate. Associations between SM invasion depth and horizontal rigidity or vertical rigidity were evaluated with Spearman’s rank sum test (r). For strength of association, an r of 0–0.10 was regarded as none, 0.10–0.39 as weak, 0.40–0.69 as moderate, 0.70–0.89 as strong, and 0.9–1 as very strong [26]. A receiver-operating characteristic (ROC) curve was drawn to estimate the area under the curve (AUC) and the most appropriate cut-off values for wall rigidity under the BE profile view to predict CRC with SM invasion depth ≥ 1.8 mm. According to the most appropriate cut-off values, test values including sensitivity, specificity, positive predictive value (PPV), negative predictive value (NPV), and accuracy were calculated. The McNemar test was used to evaluate inter-examination differences in the diagnosis of invasion depth of CRC with a cut-off value of 1.8 mm. Interobserver agreement based on kappa statistics was defined as follows: poor, 0–0.2; fair, 0.21–0.4; moderate, 0.41–0.6; substantial, 0.61–0.8; and excellent, 0.81–1. In each analysis, probabilities less than 0.05 were considered significant. For multiple comparison, Bonferroni correction was used. All statistical computations were performed with JMP version 13 (Statistical Discovery Program, Cary, NC, USA).

## Results

### Inter-observer variation in determination of radiographic or endoscopic findings

The interobserver agreement for the diagnosis of the presence of wall rigidity under the BE profile view was excellent (κ = 0.82), and that for the diagnosis of SM invasion depth ≥ 1 mm was substantial for M-NBI (κ = 0.69) and excellent for MCE (κ = 0.89).

### Association between clinicopathologic characteristics and wall rigidity in T1-CRC

The mean depth of SM invasion was 2.6 ± 1.9 mm (range: 0.1–12 mm). Fifty-three lesions had an invasion depth of less than 1.8 mm, while 92 lesions had a depth of 1.8 mm or more. Depth of SM invasion was greater for CRCs of the protruding type than the superficial type (3.3 ± 2.1 mm vs. 1.8 ± 1.4 mm, respectively; *P =* 0.0001), greater for CRCs with a microsurface of JNET type 3 than type 1/2A/2B (3.2 ± 1.8 mm vs. 2.2 ± 2.0 mm, respectively; *P =* 0.0001), and greater for CRCs of type V_I_ high-irregularity/V_N_ than type I/II/III_L_/IIIs/IV/V_I_ low-irregularity (2.9 ± 1.7 mm vs. 1.6 ± 2.4 mm, respectively; *P =* 0.0001).

Thirty CRCs did not have horizontal or vertical rigidity under the BE profile view. The mean value of horizontal rigidity was 7.2 ± 6.2 mm (range: 0–27.6 mm) while that of vertical rigidity was 2.5 ± 2.9 mm (range: 0–16 mm). As shown in Table 1, horizontal rigidity was significantly larger in the protruding type, type 3 of the JNET classification, and lesions with lympho-vascular invasion than in the superficial type, type 1/2A/2B of the JNET classification, and lesions without lympho-vascular invasion (*P* < 0.005). Horizontal and vertical rigidities were significantly different when CRCs were classified by depth of SM invasion (*P* = 0.0001). The rigidities were no different when CRCs were classified by the other clinicopathologic characteristics.

**Table 1:** Association between clinicopathological characteristics and values of wall rigidity under the barium enema examination profile view in patients with colorectal cancers(*n* = 145)

		Cases, n	Horizontal rigidity, mm	*P* value	Vertical rigidity, mm	*P* value
Tumor size	< 20 mm	91	6.0 ± 4.3	0.08	2.1 ± 2.2	0.12
	≥20 mm	54	9.1 ± 8.3		3.3 ± 3.7	
Location	Right side of the colon	38	6.2 ± 4.9	0.42	2.6 ± 2.4	0.36
	Left side of the colon	107	7.5 ± 6.6		2.5 ± 3.1	
Morphology	Protruding type	75	8.5 ± 6.2	0.002	3.2 ± 3.4	0.006
	Superficial type	70	5.7 ± 5.9		1.8 ± 2.1	
JNET classification	1/2A/2B	82	5.8 ± 6.0	0.0004	2.3 ± 3.1	0.03
	3	63	8.9 ± 6.1		2.8 ± 2.6	
Pit pattern classification	I/II/III_L_/IIIs/IV/VI low-irregularity	29	5.2 ± 6.4	0.02	2.0 ± 3.3	0.02
	VI high-irregularity/VN	116	7.7 ± 6.1		2.7 ± 2.8	
Histologic type	Well/mod/pap	143	7.1 ± 6.3	0.31	2.5 ± 2.9	0.71
	Por/sig/muc	2	9.4 ± 1.3		2.1 ± 0.8	
Depth of submucosal invasion	< 1.8 mm	53	3.2 ± 4.0	0.0001	1.2 ± 1.7	0.0001
	≥1.8 mm	92	9.4 ± 6.2		3.3 ± 3.2	
Lympho-vascular invasion	Positive	48	9.7 ± 6.8	0.0005	3.6 ± 3.8	0.008
	Negative	97	5.9 ± 5.6		2.0 ± 2.2	
Tumor budding	Grade 1	131	6.9 ± 5.9	0.25	2.5 ± 3.0	0.20
	Grade 2/3	14	10.1 ± 8.6		2.9 ± 2.2	
Lymph node metastasis	Positive	11	8.2 ± 7.0	0.76	3.0 ± 2.6	0.64
(Only lesions removed surgically)	Negative	103	8.4 ± 6.3		2.9 ± 3.1	

Continuous values are indicated as mean ± SD (standard deviation). JNET: Japan narrow-band imaging expert team

*well* well differentiated adenocarcinoma, *mod* moderately differentiated adenocarcinoma, *pap* papillary adenocarcinoma, *poor* poorly differentiated adenocarcinoma, *sig* signet-ring cell carcinoma, *muc* mucinous adenocarcinoma

### Diagnostic value of wall rigidity for the prediction of invasion depth

**Fig. 2:**
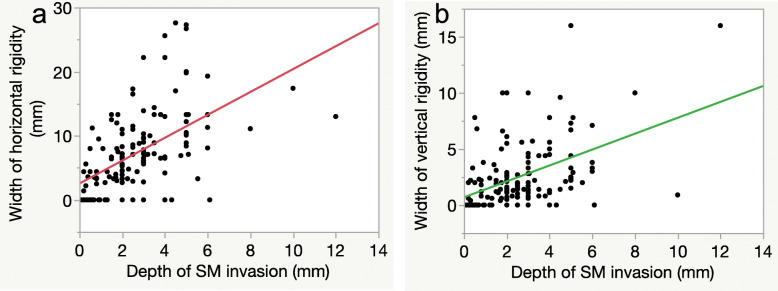
Correlation between values of wall rigidity under the barium enema examination profile view and depth of submucosal (SM) invasion. **a**: Correlation between horizontal rigidity and depth of SM invasion. The value of horizontal rigidity is moderately correlated with depth of SM invasion (y = 1.78x + 2.55, *r* = 0.626, *P* = 0.0001). **b**: Correlation between vertical rigidity and depth of SM invasion. The width of vertical rigidity is moderately correlated with depth of SM invasion (y = 0.71x + 0.69, *r* = 0.482, *P* = 0.0001)

**Fig. 3:**
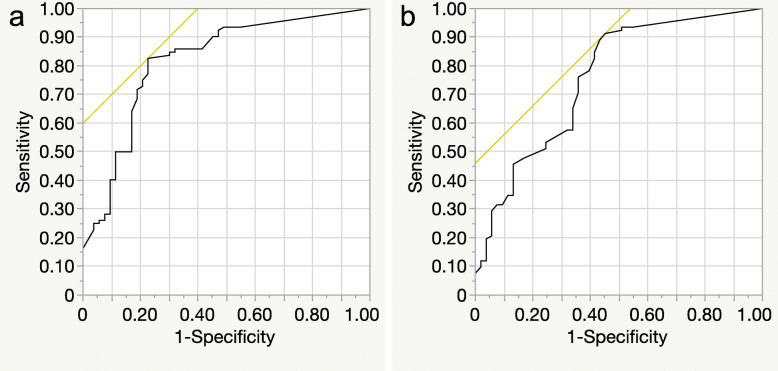
Receiver-operating characteristics curves of wall rigidity under the profile view of barium enema examination for predicting submucosal (SM) invasion depth ≥ 1.8 mm. **a**: The most appropriate cut-off value for horizontal rigidity was 4.5 mm (AUC: 0.817), with a sensitivity of 82.6%, and a specificity of 77.4%. **b**: The most appropriate cut-off value for vertical rigidity was 0.7 mm (AUC: 0.756), with a sensitivity of 91.3%, and a specificity of 54.7%

Horizontal rigidity (y = 1.78x + 2.55, *r* = 0.626, *P* = 0.0001) and vertical rigidity (y = 0.71x + 0.69, *r* = 0.482, *P* = 0.0001) showed modest correlations with the depth of SM invasion (Fig. 2). For predicting SM invasion depth ≥ 1.8 mm, the most appropriate cut-off value was 4.5 mm (AUC: 0.817), with a sensitivity of 82.6% and a specificity of 77.4% for horizontal rigidity, and it was 0.7 mm (AUC: 0.756), with a sensitivity of 91.3% and a specificity of 54.7% for vertical rigidity (Fig. 3).

### Diagnostic value of wall rigidity for the prediction of clinicopathologic characteristics

**Fig. 4:**
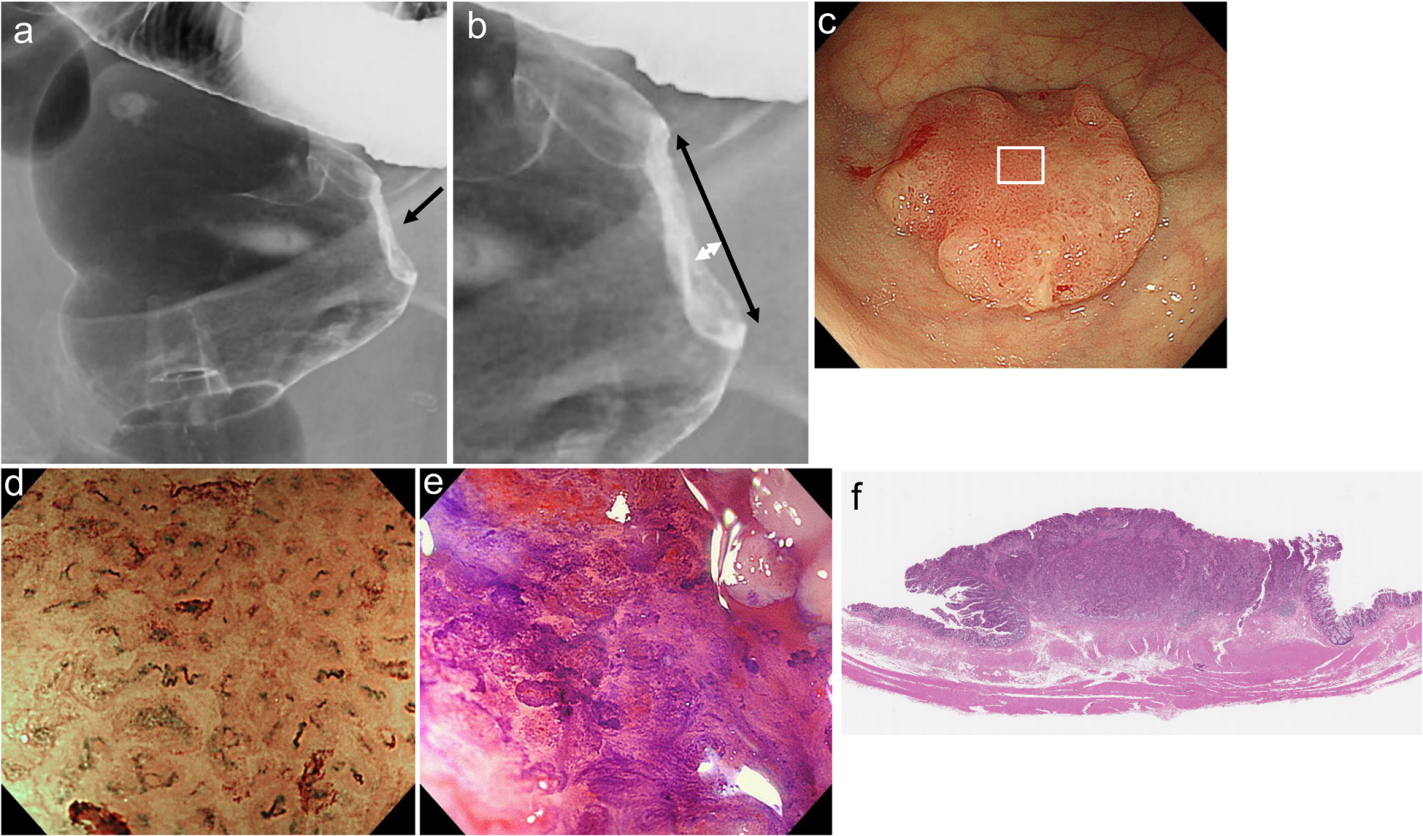
Radiographic, endoscopic and histologic features of a protruding lesion in the rectum. **a**: Barium enema (BE) examination shows a protruding lesion with an irregular depression. Wall rigidity under the BE profile view is present (arrow). **b**: High-power view of Fig. 4a. Horizontal rigidity is 17 mm (black arrow) and vertical rigidity is 1.4 mm (white arrow). **c**: Conventional colonoscopy shows a protruding lesion with a depression. **d**: Magnifying narrow-band imaging (NBI) colonoscopy for the area indicated in the box in Fig. 4c reveals loss of regular surface structure and irregular vessels. These findings are compatible with type 3 of the Japan NBI expert team classification. **e**: Magnifying chromoendoscopy with crystal violet solutions for the same area reveals an irregular and sparse surface structure, regarded as type V_N_ of the pit pattern classification. **f**: Histologic examination of the resected specimen shows a moderately differentiated adenocarcinoma invading the deep submucosal layer (invasion depth; 4.5 mm), lympho-vascular invasion-positive, budding grade 1, lymph metastasis-negative

**Fig. 5:**
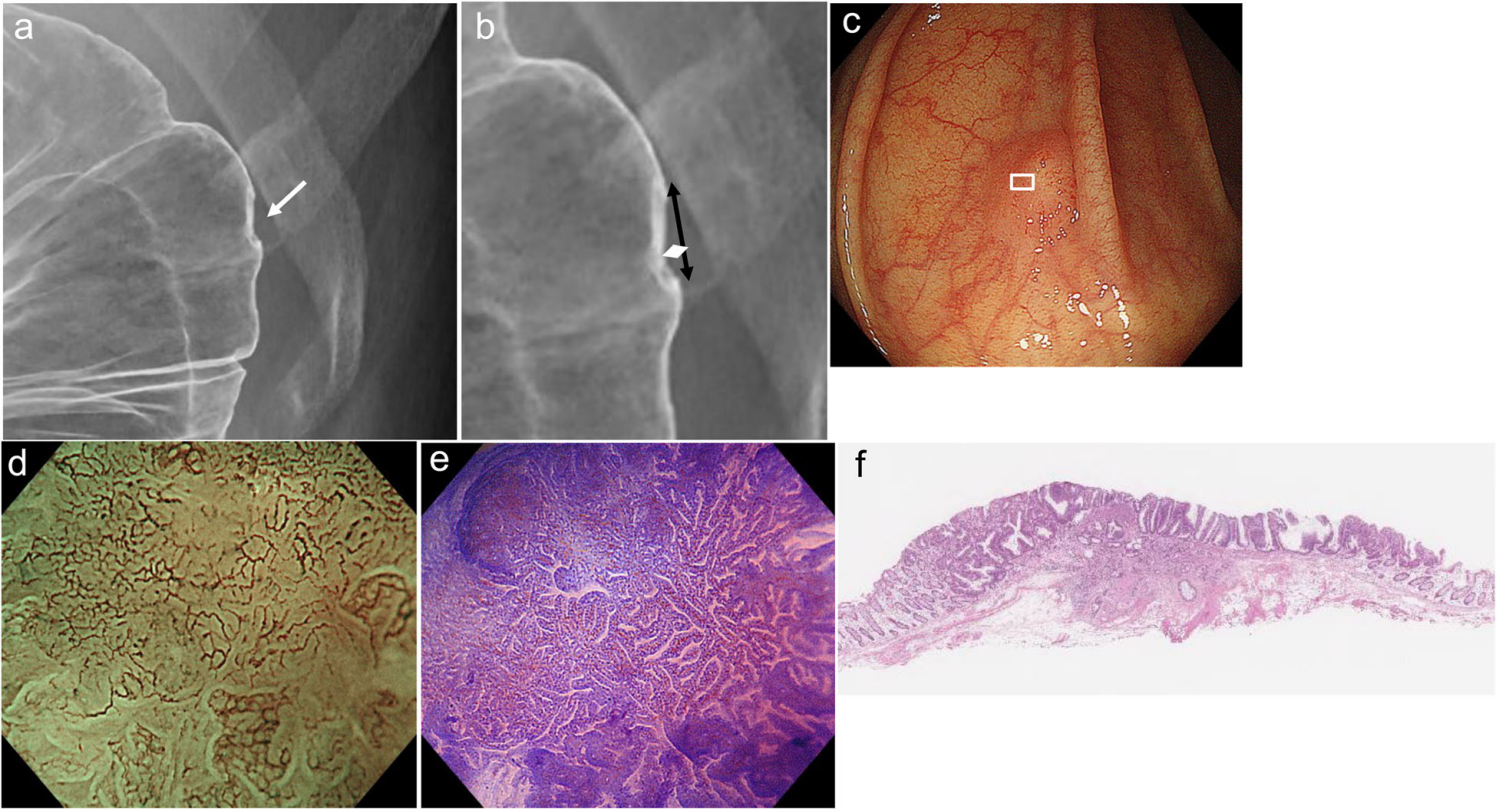
Radiographic, endoscopic and histologic features of a protruding lesion in the ascending colon. a: Barium enema (BE) examination shows a protruding lesion with an irregular depression. Wall rigidity under the BE profile view is present (arrow). b: High-power view of Fig. 5a. Horizontal rigidity is 4 mm (black arrow) and vertical rigidity is 0.4 mm (white arrow). c: Conventional colonoscopy shows a protruding lesion with a depression. d: Magnifying narrow-band imaging (NBI) colonoscopy for the area indicated in the box in Fig. 5c reveals an irregular surface structure and vessels. These findings are compatible with type 2B of the Japan NBI expert team classification. e: Magnifying chromoendoscopy with crystal violet solutions for the same area reveals an irregular surface structure, regarded as type V_I_ high-irregularity of the pit pattern classification. f: Histologic examination of the resected specimen shows a well-differentiated adenocarcinoma invading the deep submucosal layer (invasion depth; 1.7 mm), lympho-vascular invasion-negative, budding grade 1, lymph metastasis-negative

Based on the results described above, we applied cut-off values of 4.5 mm for horizontal rigidity and 0.7 mm for vertical rigidity to determine the indication for ER in CRC. When horizontal rigidity ≥4.5 mm and vertical rigidity ≥0.7 mm were defined as being positive for rigidity, 88 CRCs were positive for horizontal rigidity and 108 CRCs were positive for vertical rigidity (Fig. 4). Further, 87 CRCs were positive for both rigidities, 22 CRCs were positive for either rigidity, and 36 CRCs were negative for both rigidities (Fig. 5).

When T1-CRCs were classified by horizontal and vertical rigidities, there were significant differences in morphology, JNET classification, and lympho-vascular invasion between lesions with positive horizontal rigidity and those without (*P* < 0.005) (Table 2). There were significant differences in depth of SM invasion when lesions were classified by horizontal rigidity or vertical rigidity (*P =* 0.0001). There were no differences in the other clinicopathological characteristics between lesions classified by horizontal or vertical rigidity.

**Table 2:** Comparison of clinicopathological characteristics in widths of wall rigidity-positive lesions and -negative lesions under profile view of barium enema

		Horizontal rigidity-positive	Horizontal rigidity-negative	*P*-value	Vertical rigidity -positive	Vertical rigidity -negative	*P*-value
		(88 lesions)	(57 lesions)		(108 lesions)	(37 lesions)	
Tumor size, n (%)	< 20 mm	55 (62.5)	36 (63.2)	1.0	68 (63)	23 (62.2)	1.0
	≥20 mm	33 (37.5)	21 (36.8)		40 (37)	14 (37.8)	
Location, n (%)	Right side of the colon	22 (25)	16 (28.1)	0.70	30 (27.5)	8 (21.6)	0.52
	Left side of the colon	66 (75)	41 (71.9)		78 (72.2)	29 (78.4)	
Morphology, n (%)	Protruding type	55 (62.5)	20 (35.1)	0.002	60 (55.6)	15 (40.5)	0.13
	Superficial type	33 (37.5)	37 (64.9)		48 (44.4)	22 (59.5)	
JNET classification, n (%)	1/2A/2B	39 (44.3)	43 (75.4)	0.0003	54 (50)	28 (75.7)	0.007
	3	49 (55.7)	14 (24.6)		54 (50)	9 (24.3)	
Pit pattern classification, n (%)	I/II/III_L_/IIIs/IV/VI low- irregularity	11 (12.5)	18 (31.6)	0.01	17 (15.7)	12 (32.4)	0.03
	VI high-irregularity/VN	77 (87.5)	39 (68.4)		91 (84.3)	25 (67.6)	
Histologic type, n (%)	Well/mod/pap	86 (97.7)	57 (100)	0.52	106 (98.2)	37 (100)	1.0
	Por/sig/muc	2 (2.3)	0 (0)		2 (1.8)	0 (0)	
Depth of submucosal invasion, n (%)	< 1.8 mm	12 (13.6)	41 (71.9)	0.0001	24 (22.2)	29 (78.4)	0.0001
	≥1.8 mm	76 (86.4)	16 (28.1)		84 (77.8)	8 (21.6)	
Lympho-vascular invasion, n (%)	Positive	38 (43.2)	10 (17.5)	0.002	41 (38)	7 (18.9)	0.03
	Negative	50 (56.8)	47 (82.5)		67 (62)	30 (81.1)	
Tumor budding, n (%)	Grade 1	78 (88.6)	53 (93)	0.57	96 (88.9)	35 (94.6)	0.52
	Grade 2/3	10 (11.4)	4 (7)		12 (11.1)	2 (5.4)	
Lymph node metastasis, n (%)	Positive	7 (8.6)	4 (12.1)	0.73	9 (9.6)	2 (10)	1.0
(Only lesions removed surgically)	Negative	74 (91.4)	29 (87.9)		85 (90.4)	18 (90)	

Horizontal rigidity-positive is defined as horizontal rigidity ≥4.5 mm. Vertical rigidity-positive is defined as vertical rigidity ≥0.7 mm

Continuous values are indicated as mean ± SD (standard deviation). JNET: Japan narrow-band imaging expert team

*well* well differentiated adenocarcinoma, *mod* moderately differentiated adenocarcinoma, *pap* papillary adenocarcinoma, *poor* poorly differentiated adenocarcinoma, *sig* signet-ring cell carcinoma, *muc* mucinous adenocarcinoma

### Diagnostic ability of horizontal and vertical rigidities under BE for the diagnosis of SM invasion depth ≥ 1.8 mm

As shown in Table 3, the diagnostic test results of the cut-off values of 4.5 mm for horizontal rigidity and 0.7 mm for vertical rigidity were statistically significantly different (*P =* 0.01).

**Table 3:** Comparison of the diagnostic ability for submucosal invasion depth ≥ 1.8 mm between horizontal and vertical rigidities under the barium enema examination profile view

145 lesions		Vertical rigidity (≥0.7 mm)	
Horizontal rigidity (≥4.5 mm)		Positive	Negative
	Positive	87	1
	Negative	21	36
*χ*2		16.41	
*P* value		0.01	

When T1-CRCs were classified into three groups according to rigidity, SM invasion depth ≥ 1.8 mm was most common in lesions positive for both rigidities (86.2%), followed by lesions positive for any one rigidity (45.5%) and those negative for both rigidities (19.4%) (*P* = 0.0001) (Table 4). The incidence of SM invasion ≥1.8 mm was significantly higher in lesions positive for both rigidities than in those positive for either one rigidity (*P* = 0.0006) and in those negative for both rigidities (*P* = 0.0003).

**Table 4:** Comparison of the incidence of colorectal cancers with submucosal invasion depth ≥ 1.8 mm between the combinations of horizontal and vertical rigidities under the barium enema examination profile view

145 lesions		Positive for horizontal and vertical rigidity (*n* = 87)	Positive for either rigidity (*n* = 22)	Negative for both rigidities (*n* = 36)	*P* value
Depth of submucosal invasion, n (%)	< 1.8 mm	12 (13.8)	12 (54.5)	29 (80.6)	0.0001
	≥1.8 mm	75 (86.2)*^†^	10 (45.5)*	7 (19.4)^†^	

Positive for horizontal rigidity is defined as horizontal rigidity ≥4.5 mm. Positive for vertical rigidity is defined as vertical rigidity ≥0.7 mm

*P = 0.0006, ^†^*P* = 0.0003

As shown in Table 5, the specificity, PPV and accuracy of horizontal rigidity and the combination of the rigidities for the diagnosis of SM invasion depth ≥ 1.8 mm were higher than those of vertical rigidity. In contrast, the sensitivity and NPV of vertical rigidity were higher than those of horizontal rigidity and the combination of the rigidities.

**Table 5:** Comparison of diagnostic test results of horizontal and vertical rigidities under the barium enema examination profile view for predicting submucosal invasion depth ≥ 1.8 mm

	Sensitivity (%)	Specificity (%)	PPV (%)	NPV (%)	Accuracy (%)
Horizontal rigidity ≥4.5 mm	82.6	77.4	86.4	71.9	80.7
Vertical rigidity ≥0.7 mm	91.3	54.7	77.8	78.4	77.9
Horizontal rigidity ≥4.5 mm and vertical rigidity ≥0.7 mm	81.5	77.4	86.2	70.1	80.0

*PPV* positive predictive value, *NPV* negative predictive value

## Discussion

In the present study, the values of horizontal and vertical rigidity under the BE profile view were correlated with the depth of SM invasion, and the horizontal rigidity was greater in lympho-vascular invasion-positive lesions than in -negative lesions. Using the most appropriate cut-off values of wall rigidity for predicting SM invasion depth ≥ 1.8 mm, we showed that the horizontal rigidity was predictive of lympho-vascular invasion and that the two rigidities were independent of each other for the diagnosis of invasion depth in CRCs. These observations suggest that rigidity under the BE profile view may be useful for making therapeutic decisions in T1-CRC.

In the 2019 JSCCR guidelines, surgical resection is recommended for clinical T1b (SM invasion depth ≥ 1 mm), because the rate of lymph node metastasis for CRCs with an SM invasion ≥1 mm was higher (12.5%) than that for CRCs with an SM invasion < 1 mm (1.6%) [3, 27]. It has been reported that fold convergency, smooth surface, irregularity in depression, deep depression, and wall rigidity on the profile view are suggestive of SM invasion depth ≥ 1 mm [13, 22]. Among these BE findings, wall rigidity on the profile view is a specific finding that cannot be depicted by endoscopy. Wall rigidity under the BE profile view has been shown to be a consequence of poor colorectal distension due to fibrosis associated with submucosal or deeper infiltration of carcinoma cells [13, 28, 29]. To date, however, no study has shown a correlation between wall rigidity under the BE profile view and invasion depth in T1-CRC. Our results showed that horizontal and vertical rigidity are modest signs for the diagnosis of SM invasion depth in CRC. Although we were not able to quantify the pathologic findings in the present study, it can be presumed that the horizontal and vertical rigidity reflect the volume of fibrosis associated with cancer infiltration.

For the diagnosis of SM invasion depth ≥ 1 mm under BE, Watari et al. [13] reported that the accuracy of radiographic findings, such as fold convergency, eccentric deformity on the profile view, deep central depression, irregularity in depression and tumor size > 20 mm, was 85% in cases positive for at least one of those radiographic findings. Hisabe et al. [30] showed that the accuracy of the enface rigidity under BE for the diagnosis of SM invasion ≥1 mm was 82% for the protruding type and 78.8% for the superficial type. Those prior studies evaluated the value of BE for the distinction of SM invasion depth ≥ 1 mm and < 1 mm. Although SM invasion depth ≥ 1 mm is a pathologic risk factor for lymph node metastasis, it has been shown that the rate of lymph node metastasis was only 1–2% in T1-CRCs negative for the other risk factors, although the SM invasion depth was greater than 1 mm [9, 31, 32]. Furthermore, Nakadoi et al. [5] showed that T1-CRCs with SM invasion depth < 1.8 mm without the other risk factors were completely free from lymph node metastasis. Based on this observation, we applied a cut-off value of 1.8 mm in this study. Consequently, our study showed that horizontal rigidity may be predictive of lympho-vascular invasion. However, it should also be noted that 17.5% of CRCs negative for horizontal rigidity actually had lympho-vascular invasion. These results suggest that the rigidities under BE may be useful for the prediction of lympho-vascular invasion, but they should not be regarded as absolute markers.

Our results indicated that there was a statistically significant difference in the diagnostic value between horizontal rigidity and vertical rigidity. This observation suggests that each rigidity is independent of the other in the interpretation of BE findings. In addition, the prevalence of lympho-vascular invasion was different when lesions were classified by horizontal rigidity, and, furthermore, the rate of SM invasion depth ≥ 1.8 mm was statistically significantly different when lesions were classified into three groups according to the combination of horizontal and vertical rigidity. We thus believe that both rigidities should be carefully and independently assessed for the diagnosis of invasion depth when BE is to be applied to T1-CRC.

Studies of three-dimensional computed tomography air-contrast enema (CT enema) simulating BE have been reported [28, 29, 33–35]. Also, computer-aided diagnosis systems including artificial intelligence (AI) have been developed [36, 37]. Several studies have measured the wall rigidity under the profile view of the CT enema in CRC. In a previous study, vertical rigidity in a cross-sectional multiplanar reconstruction image was found to have a sensitivity of 90.5% and a specificity of 100% for differentiating T1-CRC from high-grade dysplasia [29]. In other studies, a combination of the length of the deformity and the angle of the deformed outline correlated significantly with the invasion depth of CRCs, with an accuracy of 88.7% for the diagnosis of T1-CRC [28, 33, 38]. Thus, we can apply wall rigidity under BE to CT enema, and we may also be able to improve the diagnostic ability of AI-assisted CT enema with deep learning by understanding wall rigidity under BE.

The present study has several limitations. First, because we studied T1-CRCs that were evaluated by BE, M-NBI, and MCE, we may have evaluated CRCs in which the diagnosis of invasion depth was extremely difficult. It thus seems possible that the subject lesions of our study may not have been representative of overall T1-CRCs. Second, because this was a retrospective study, we could not control for heterogeneity in the quality of BE and colonoscopic images. However, this limitation does not preclude the application of our findings in the clinical setting. Third and most significantly, the use of BE is decreasing. However, our findings appear significant when AI-assisted CT enema can be applied for the assessment of wall rigidity. The cut-off value of 4.5 mm for horizontal rigidity in this study may be able to be applied to CT enema. In contrast, the limited vertical rigidity in this study may be not applicable to CT enema when the overall spatial resolution of CT is taken into consideration. Finally, we could not show data of inter-observer variation or a second independent validation cohort to validate most appropriate cut-off value for the rigidity, because the number of T1-CRCs in our retrospective cohort was small. It thus is necessary to validate our observations in a prospective cohort.

## Conclusions

Our study revealed that the horizontal and vertical rigidities under a BE profile view were correlated with SM invasion depth. Using the cut-off values for predicting SM invasion depth ≥ 1.8 mm, horizontal rigidity may be predictive of lympho-vascular invasion. These findings may be helpful for decision making in the treatment of T1-CRCs.

## Data Availability

Not applicable.

## Abbreviations

ER: Endoscopic resection
ESD: Endoscopic submucosal dissection
EMR: Endoscopic mucosal resection
CRC: Colorectal cancer
T1-CRC: Submucosally invasive colorectal cancer
SM: Submucosal
JSCCR: The Japanese Society for Cancer of the Colon and Rectum
BE: Barium enema examination
M-NBI: Magnifying narrow-band imaging colonoscopy
MCE: Magnifying chromoendoscopy
JNET: Japan narrow-band imaging Expert Team
SD: Standard deviation
ROC: Receiver-operating characteristic
AUC: Area under the curve
PPV: Positive predictive value
NPV: Negative predictive value
CT enema: Computed tomography air-contrast enema
AI: Artificial intelligence
well: well-differentiated adenocarcinoma
mod: moderately differentiated adenocarcinoma
pap: papillary adenocarcinoma
poor: poorly differentiated adenocarcinoma
sig: signet-ring cell carcinoma
muc: mucinous adenocarcinoma
